# Shared and distinct dynamic brain activity and functional connectivity variability in patients with isolated craniocervical dystonia

**DOI:** 10.1093/braincomms/fcag122

**Published:** 2026-04-02

**Authors:** Jiatai Lin, Jiana Zhang, Zhengkun Yang, HaiLiang Shen, Linchang Zhong, Huiming Liu, Zilin Ou, Zhicong Yan, Weixi Zhang, Kangqiang Peng, Gang Liu, Jinping Xu

**Affiliations:** Department of Biomedical Engineering, Southern University of Science and Technology, Shenzhen 518055, China; Institute of Biomedical and Health Engineering, Shenzhen Institutes of Advanced Technology, Chinese Academy of Sciences, Shenzhen 518055, China; Department of Biomedical Engineering, Shenzhen University of Advanced Technology, Shenzhen 518107, China; Department of Neurology, The First Affiliated Hospital, Sun Yat-sen University, Guangdong Provincial Key Laboratory for Diagnosis and Treatment of Major Neurological Diseases, National Key Clinical Department and Key Discipline of Neurology, Guangzhou 510080, China; Department of Neurology, The First Affiliated Hospital, Sun Yat-sen University, Guangdong Provincial Key Laboratory for Diagnosis and Treatment of Major Neurological Diseases, National Key Clinical Department and Key Discipline of Neurology, Guangzhou 510080, China; Department of Biomedical Engineering, Southern University of Science and Technology, Shenzhen 518055, China; Institute of Biomedical and Health Engineering, Shenzhen Institutes of Advanced Technology, Chinese Academy of Sciences, Shenzhen 518055, China; Department of Biomedical Engineering, Shenzhen University of Advanced Technology, Shenzhen 518107, China; Department of Medical Imaging, Sun Yat-Sen University Cancer Center, State Key Laboratory of Oncology in Southern China, Collaborative Innovation Center for Cancer Medicine, Guangzhou 510060, China; Department of Medical Imaging, Sun Yat-Sen University Cancer Center, State Key Laboratory of Oncology in Southern China, Collaborative Innovation Center for Cancer Medicine, Guangzhou 510060, China; Department of Neurology, The First Affiliated Hospital, Sun Yat-sen University, Guangdong Provincial Key Laboratory for Diagnosis and Treatment of Major Neurological Diseases, National Key Clinical Department and Key Discipline of Neurology, Guangzhou 510080, China; Department of Neurology, The First Affiliated Hospital, Sun Yat-sen University, Guangdong Provincial Key Laboratory for Diagnosis and Treatment of Major Neurological Diseases, National Key Clinical Department and Key Discipline of Neurology, Guangzhou 510080, China; Department of Neurology, The First Affiliated Hospital, Sun Yat-sen University, Guangdong Provincial Key Laboratory for Diagnosis and Treatment of Major Neurological Diseases, National Key Clinical Department and Key Discipline of Neurology, Guangzhou 510080, China; Department of Medical Imaging, Sun Yat-Sen University Cancer Center, State Key Laboratory of Oncology in Southern China, Collaborative Innovation Center for Cancer Medicine, Guangzhou 510060, China; Department of Neurology, The First Affiliated Hospital, Sun Yat-sen University, Guangdong Provincial Key Laboratory for Diagnosis and Treatment of Major Neurological Diseases, National Key Clinical Department and Key Discipline of Neurology, Guangzhou 510080, China; Guangdong-HongKong-Macao Greater Bay Area Center for Brain Science and Brain-Inspired Intelligence, Guangzhou 510515, China; Institute of Biomedical and Health Engineering, Shenzhen Institutes of Advanced Technology, Chinese Academy of Sciences, Shenzhen 518055, China

**Keywords:** dystonia, dynamic amplitude of low-frequency fluctuations, dynamic functional connectivity, dynamic regional homogeneity, cerebellum

## Abstract

Isolated craniocervical dystonia is a hyperkinetic movement disorder with motor and non-motor features, yet the temporal dynamics of its brain activity remain underexplored. Using resting-state functional MRI from 201 isolated craniocervical dystonia patients (102 blepharospasm, 43 blepharospasm–oromandibular dystonia and 56 cervical dystonia) and 160 healthy controls, we applied a sliding-window approach to evaluate dynamic functional metrics and dynamic functional connectivity patterns. Isolated craniocervical dystonia patients showed widespread abnormalities in dynamic regional activity involving the cerebellum, primary motor cortex and visual cortex, which were consistent with instability in neural activity or coordination. Subnetwork analyses indicated significant alterations—particularly increased dynamic functional connectivity variability in cerebello-cortical and cortico-cortical circuits—in isolated craniocervical dystonia and blepharospasm as compared to healthy controls. Clustering of time-resolved connectivity revealed two recurring brain states: a strongly connected State 1 and a weakly connected State 2. Compared to healthy controls, isolated craniocervical dystonia, blepharospasm, blepharospasm–oromandibular dystonia and cervical dystonia groups spent proportionally more time in State 2 and exhibited fewer state transitions, suggesting reduced network flexibility and a bias toward hypo-connected configurations. Together, these findings demonstrated shared and subtype-specific disruptions in the dynamics of spontaneous activity and interregional coupling in isolated craniocervical dystonia, implicating alterations of cerebello-cortical and sensorimotor-visual systems in its pathophysiology and highlighting temporal instability and diminished switching as potential disease signatures.

## Teaser

Lin *et al*. found that isolated dystonia patients show unstable cerebellar, motor and visual cortex activity with increased functional connectivity variability. They exhibit two brain states, spending more time in a weakly connected state with fewer transitions, indicating reduced network flexibility and temporal instability.

## Graphical Abstract

**Graphical Abstract:**
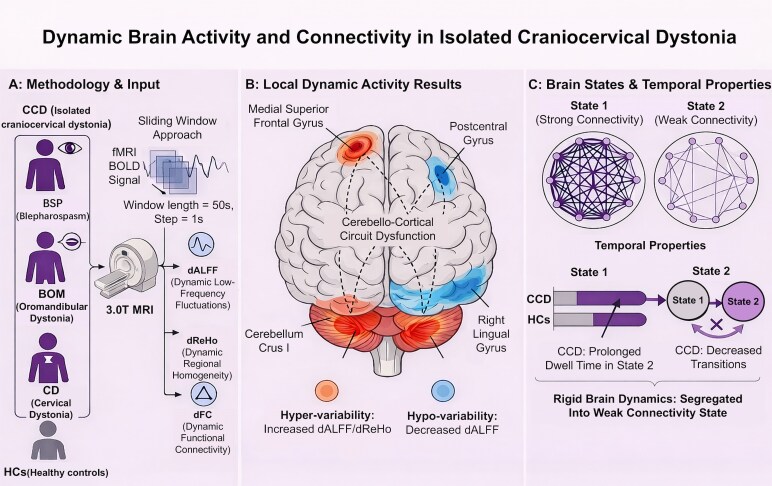

## Introduction

Isolated craniocervical dystonia (CCD) is a neurological disorder characterized by abnormal motor control, with involuntary muscle contractions causing twisting movements and abnormal postures.^1^ Common types include blepharospasm (BSP), blepharospasm-oromandibular dystonia (BOM) and cervical dystonia (CD). Studies have shown that BSP, BOM and CD often exhibit clinical continuity and co-occurrence. For instance, in Meige syndrome, some BSP patients may progressively develop oromandibular involvement and further extend to the neck, manifesting as CCD.^2,3^ However, the shared and distinct underlying aetiology and pathophysiology of BSP, BOM and CD are not fully understood.

Functional magnetic resonance imaging (fMRI), as a non-invasive tool, has revealed abnormal activity in multiple brain regions in patients with BSP, BOM and CD through both task-specific and resting-state fMRI studies. Task-specific fMRI studies have shown that compared to healthy controls (HCs), BSP patients exhibited stronger activation in the visual cortex, primary motor cortex and cerebellum during active blinking, BOM patients showed reduced activation in the primary sensorimotor cortex, premotor cortex and sensory association areas during vocalization, and CD patients showed activation in the bilateral basal ganglia, contralateral cerebellum and sensorimotor cortex during an isometric wrist extension task.^4,5^ Consistent with these findings, other neuroimaging studies indicated that patients with BSP, BOM and CD exhibited abnormal spontaneous brain activity in specific regions. There are common abnormalities as well as some disease-specific differences, which are reflected through amplitude of low-frequency fluctuations (ALFF), regional homogeneity (ReHo) and resting-state functional connectivity (FC). These abnormalities were involved in multiple neural networks, such as the cerebellum, default mode network, cortico-basal ganglia-thalamic loop, sensorimotor network, visual network, fronto-parietal network and salience network.^4,6-10^ However, these studies were performed based on the assumption that brain activity remains static during fMRI scanning, overlooking the fact that neural activity fluctuates dynamically over time.^11^ Recent research based on task-specific fMRI and electrophysiological studies has provided evidence suggesting that functional activity and connectivity may change dynamically within seconds to minutes.^12^ In light of this, dynamic fMRI analysis methods have been proposed. Applying dynamic brain network analysis to CCD patients can provide finer and more informative insights, revealing the processes of neural alterations as well as the functional coordination and adaptation of brain networks,^8,13^ thereby further uncovering time-varying evidence of abnormal brain function in CCD patients.

Regional homogeneity (ReHo) reflects the local synchronization of a given voxel with its neighbouring voxels.^14^ ALFF measures the regional intensity of spontaneous fluctuations within an effective frequency range (0.01–0.08 Hz), representing the power of spontaneous brain activity.^15^ FC reflects the neural signal correlation between two brain regions.^16^ By integrating ALFF, ReHo and FC measurements with the sliding window method, dynamic ALFF (dALFF), dynamic ReHo (dReHo) and dynamic FC (dFC) were introduced to capture the temporal variance of ALFF, ReHo and FC.^17-19^ These approaches have been applied to various types of isolated dystonia^8,9,20^ and other movement disorders.^21,22^ These results revealed valuable information overlooked by traditional static methods,^23^ thus offering deeper insights into the mechanisms of brain activity and connectivity.^24^ However, the similarities and differences in dynamic characteristics and temporal properties among different subtypes of CCD remain unclear.

Therefore, the aim of this study was to characterize the shared and subtype-specific patterns of dynamic brain activity and dFC variability in CCD and its subtypes. We hypothesized that CCD subtypes would show common abnormalities in the temporal variability of brain activity and connectivity, while exhibiting subtype-specific differences.

## Methods and materials

### Participants

This study was approved by the Ethics Committee of the First Affiliated Hospital of Sun Yat-sen University ([2020]323), and all participants signed written informed consent in accordance with the Declaration of Helsinki. Patients with CCD were recruited from the movement disorders outpatient clinic. Patients were excluded if they met any of the following criteria: (i) aged under 18 years, (ii) acquired or functional dystonia, (iii) severe psychiatric disorders or cognitive impairment, (iv) other neurological diseases besides dystonia (such as stroke, epilepsy, traumatic brain injury, brainstem demyelination, encephalitis, or brain hypoxia), (v) received botulinum toxin (BoNT) injections within 3 months before the MRI, related medical treatments, or surgery within the 3 months prior to the MRI scan, (vi) contraindications to MRI. Ultimately, 102 patients with BSP, 43 patients with BOM and 56 patients with CD were included in this study. We also recruited 160 HCs.

### Clinical assessment

Each participant underwent a face-to-face interview before the scan to collect demographic and clinical data, including age, gender, disease duration, disease severity and the timing of BoNT injections. Disease severity in BSP patients was assessed using the Jankovic Rating Scale (JRS), which includes two subscales to evaluate the severity and frequency of involuntary orbicularis oculi muscle contractions.^25^ Disease severity in BOM patients was assessed using the Burke-Fahn-Marsden Dystonia Rating Scale (BFMDRS),^26^ while disease severity in CD patients was assessed using the Toronto Western Spasmodic Torticollis Rating Scale (TWSTRS).^27^

### fMRI data acquisition

Imaging data were acquired using a 3T scanner (Tim Trio; Siemens). During resting-state fMRI scanning, participants were instructed to keep their eyes closed, remain still with their heads fixed, and stay awake. Resting-state fMRI data were collected using an echo-planar imaging (EPI) sequence with the following parameters: repetition time (TR) = 2000 ms, echo time (TE) = 30 ms, field of view (FOV) = 220 × 220 mm^2^, flip angle = 90°, voxel size = 3.44 × 3.44 × 3 mm^3^, averages = 1 and 33 slices.

### fMRI data pre-processing

Resting-state fMRI data were processed using the Data Processing and Analysis of Brain Imaging (DPABI) toolbox (http://rfmri.org/dpabi).^28^ The preprocessing pipeline comprised the following steps: (i) discarding the first 10 volumes to ensure signal stabilization and allow participants to acclimate to the scanning environment; (ii) slice timing correction and head motion correction; (iii) normalizing to the Montreal Neurological Institute (MNI) space using the EPI template image; (iv) resampling to a voxel size of 3 × 3 × 3 mm^3^; (v) spatial smoothing with a Gaussian kernel of 6-mm full-width at half-maximum; (vi) detrending; (vii) noise removal, including regression of Friston-24 head motion parameters, cerebrospinal fluid signals and white matter signals; (viii) bandpass filtering (0.01–0.1 Hz). Given the ongoing debate about the use of global signal regression in resting-state fMRI, concerns regarding its potential to contain meaningful neuronal information, and the risk that global signal regression might introduce spurious results in seed-based voxel correlation analyses,^29^ this step was omitted.

### dALFF and dReHo variability analysis

**Figure 1:**
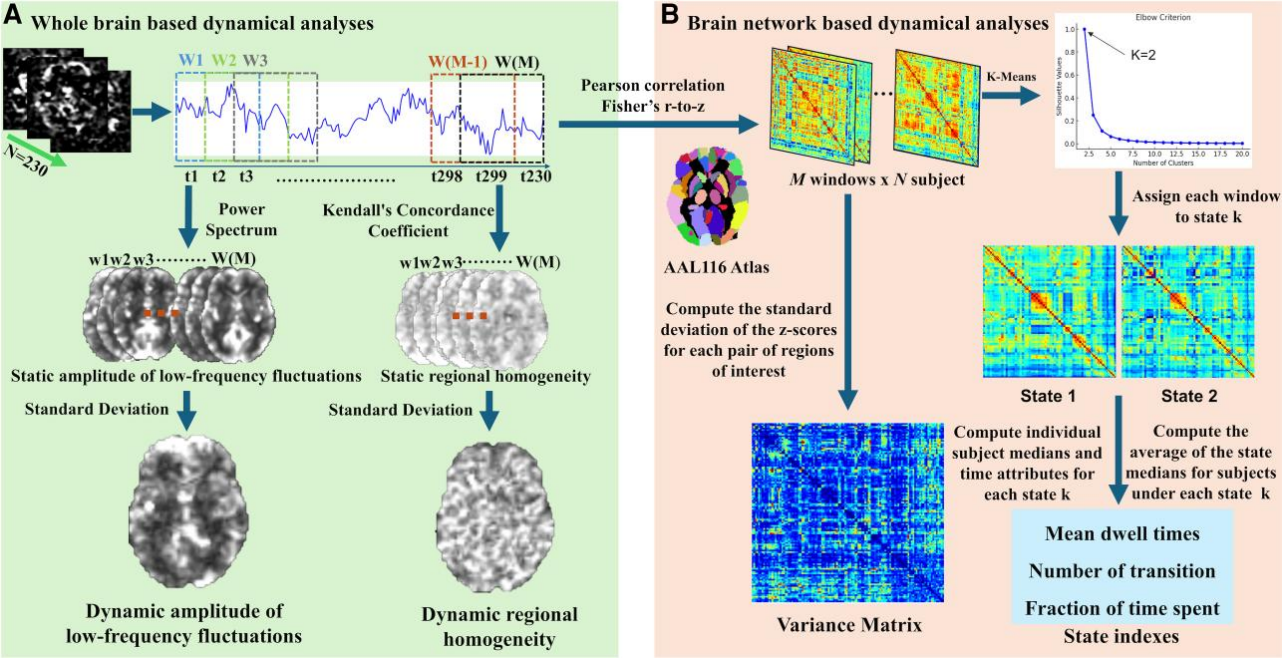
**Analysis pipeline for brain activity and functional connectivity.** The pipeline includes two steps: (**A**) Using a sliding-window approach to compute dALFF and dReHo to assess temporal variability of regional spontaneous brain activity; (**B**) calculating variability of dFC matrices via a sliding-window approach, followed by K-means clustering to classify each time window’s connectivity pattern into one of two dynamic brain network states, with subsequent computation of dynamic connectivity strength and temporal properties for each subject in each state.

dALFF and dReHo were analysed (Fig. 1) using the sliding window method, employing the Time Dynamic Analysis (TDA) toolbox in DPABI. Previous studies have shown that the choice of sliding window length can influence the results, although no standardized criteria have been established.^30^ To minimize the potential for spurious fluctuations, the minimum window length was set to exceed 1/f_min, where f_min denotes the lowest frequency in the time series.^31^ Based on previous research, a sliding window of 50 s in length with a step size of 1 s was used to assess the variability of global dALFF and dReHo.^8,32,33^ As a result, each subject's 230 time points were divided into 181 overlapping windows. For each sliding window, ALFF and ReHo were calculated for each voxel. ALFF is calculated by transforming each voxel's time series into the frequency domain using a fast Fourier transform (FFT), and then the square root of the power spectrum within the 0.01–0.08 Hz range was calculated to obtain each voxel's ALFF value, and averaged within this frequency band. ReHo was calculated by determining the Kendall’s coefficient of concordance for the time series of the 27 nearest neighbouring voxels for each voxel.^34^ To assess temporal variability, the standard deviation (SD) of the ALFF and ReHo values for each voxel across the 181 windows was calculated, yielding the dALFF and dReHo variability maps. These maps were then standardized into *z*-scores to enhance the normality of their distribution. To investigate the potential clinical relevance of the functional metrics, significant clusters identified from the voxel-wise comparison between the CCD group and HCs were defined as regions of interest (ROIs). Mean dALFF and dReHo variability values within these ROIs were extracted for each participant and used for subsequent statistical analysis.

### Dynamic FC estimation and variability analysis

Dynamic FC analysis was performed using the sliding time window technique from the Dynamic BC toolbox.^35^ Based on the automated anatomical labelling (AAL) template with 116 regions, 116 ROIs were generated, with the first 90 regions (AAL1-90) corresponding to the cerebrum and the remaining 26 regions (AAL91-116) corresponding to the cerebellum.^36^ The sliding window method was set with a window size of 50 s and a step size of 1 s, generating 181 overlapping windows for each participant. Within each window, the Pearson correlation coefficient between all pairwise ROI time series was calculated, resulting in a 116 × 116 FC matrix. To improve the normality of the correlation distribution, Fisher’s *r*-to-*z* transformation was applied to convert the correlation coefficients into *z*-values. To assess the variability of dFC, the SD of the *z*-values for each pair of ROIs was calculated, generating the FC strength variability matrix, reflecting the connectivity variability between ROIs over time.

### Clustering and state analysis

Based on previous studies,^37^ we applied the K-means algorithm to the FC matrices of all subject windows to identify recurring FC patterns, thus revealing the brain's FC states at different time points. We set the number of clusters to 6, randomly initialized the cluster centres, and performed 500 clustering iterations. The optimal number of clusters was determined using the silhouette coefficient algorithm, which calculates the similarity of a point to other points in the same cluster and compares it to the similarity with points in other clusters.^38^ Ultimately, the best number of clusters was determined to be 2, and all participants’ FC matrices were classified into two states based on their similarity to the cluster centres.^39^ To visualize the group-specific cluster centres, we calculated the average value of the subject-specific cluster centres for each group and also performed two-sample *t*-tests to compare the connectivity strength of each state between the HCs and CCD\BSP\BOM\CD (*P* < 0.01, top 0.4%). Additionally, we calculated the mean dwell time (MDT), the proportion of dwell time and the number of transitions for each state to investigate the temporal properties of dFC. MDT reflects the average duration of each state, derived by calculating the average number of consecutive windows belonging to the same state. The proportion of dwell time indicates the proportion of total time spent in each state, while the number of transitions refers to the frequency of transitions between different FC states.

### Statistical analyses

Statistical analyses were performed using Python. Demographic and clinical data were analysed with non-parametric tests according to their distribution. Group differences in neuroimaging metrics (dALFF, dReHo, dFC variability) and state temporal properties were assessed using two-sample *t*-tests, with age and sex included as covariates. Multiple comparison corrections were applied according to the type of measure: voxel-wise regional metrics were corrected using Gaussian random field (GRF) theory (voxel-level *P* < 0.01, cluster-level *P* < 0.05); dFC variability was corrected using Network-Based Statistic (NBS) (edge threshold *P* < 0.005, 1000 permutations, component *P* < 0.05); temporal properties of states and *post hoc* ROI analyses were corrected using false discovery rate (FDR; *P* < 0.05). In addition, Spearman partial correlation analyses were conducted to evaluate the relationships between imaging metrics in significant ROIs and clinical characteristics.

## Result

### Demographic information and clinical characteristics

As shown in Table 1, there were no significant differences in age and sex between the CCD and HCs. The BSP, BOM, CD and HCs groups showed no significant differences in sex, but significant differences in age. No significant differences were found in BoNT treatment history among the BSP, BOM and CD groups. The BSP and BOM groups exhibited no significant differences in JRS frequency subscore and JRS total score, but significant differences were observed in JRS severity subscore and disease duration.

**Table 1:** Demographic and clinical data of study participants

		CCD (*n* = 201)		HCs (*n* = 160)	*χ^2^/z*-value	*P*–value
Age (years)		52.59 ± 14.67		47.91 ± 13.27	−1.744	0.081
Sex (male/female)		71/130		57/103	0	1.000^a^
	BSP (*n* = 102)	BOM (*n* = 43)	CD (*n* = 56)	HCs (*n* = 160)	*H/χ^2^/z*-value	*P*-value
Age (years)	53.70 ± 9.02	56.26 ± 10.92	40.34 ± 11.72	47.91 ± 13.27	53.549	<0.001^b^
Sex (male/female)	31/71	14/29	26/30	57/103	4.25	0.236^a^
Duration (years)	3.29 ± 3.62	4.88 ± 4.36	3.07 ± 3.78	—	12.055	0.002^b^
Duration of BoNT treatment history (years)	2.71 ± 2.66 (45)	2.76 ± 2.63 (22)	2.71 ± 2.35 (13)	—	2.784	0.249^b^
JRS severity subscore	3.16 ± 0.66	3.35 ± 0.97	—	—	−2.454	0.014^b^
JRS frequency subscore	3.01 ± 0.70	2.88 ± 0.88	—	—	−0.581	0.561^b^
JRS total score	6.17 ± 1.17	6.23 ± 1.73	—	—	−1.599	0.110^b^
BFMDRS	—	7.33 ± 2.30	—	—	—	—
TWSTRS total score	—	—	35.26 ± 7.80 (48)	—	—	—

Abbreviations: BoNT, botulinum toxin; CCD, isolated craniocervical dystonia; BSP, blepharospasm; HCs, healthy controls; CD, craniocervical dystonia; BOM, blepharospasm-oromandibular dystonia; JRS, Jankovic Rating Scale; BFMDRS, Burke-Fahn-Marsden Dystonia Rating Scale ;TWSTRS, The Toronto Western Spasmodic Torticollis Rating Scale.

^a^Kruskal–Wallis *H*-test used.

^b^Chi-square test used.

^c^Mann–Whitney U-test used. The number of participants is indicated in parentheses.

### Between-group differences in dALFF and dReHo variability

**Figure 2:**
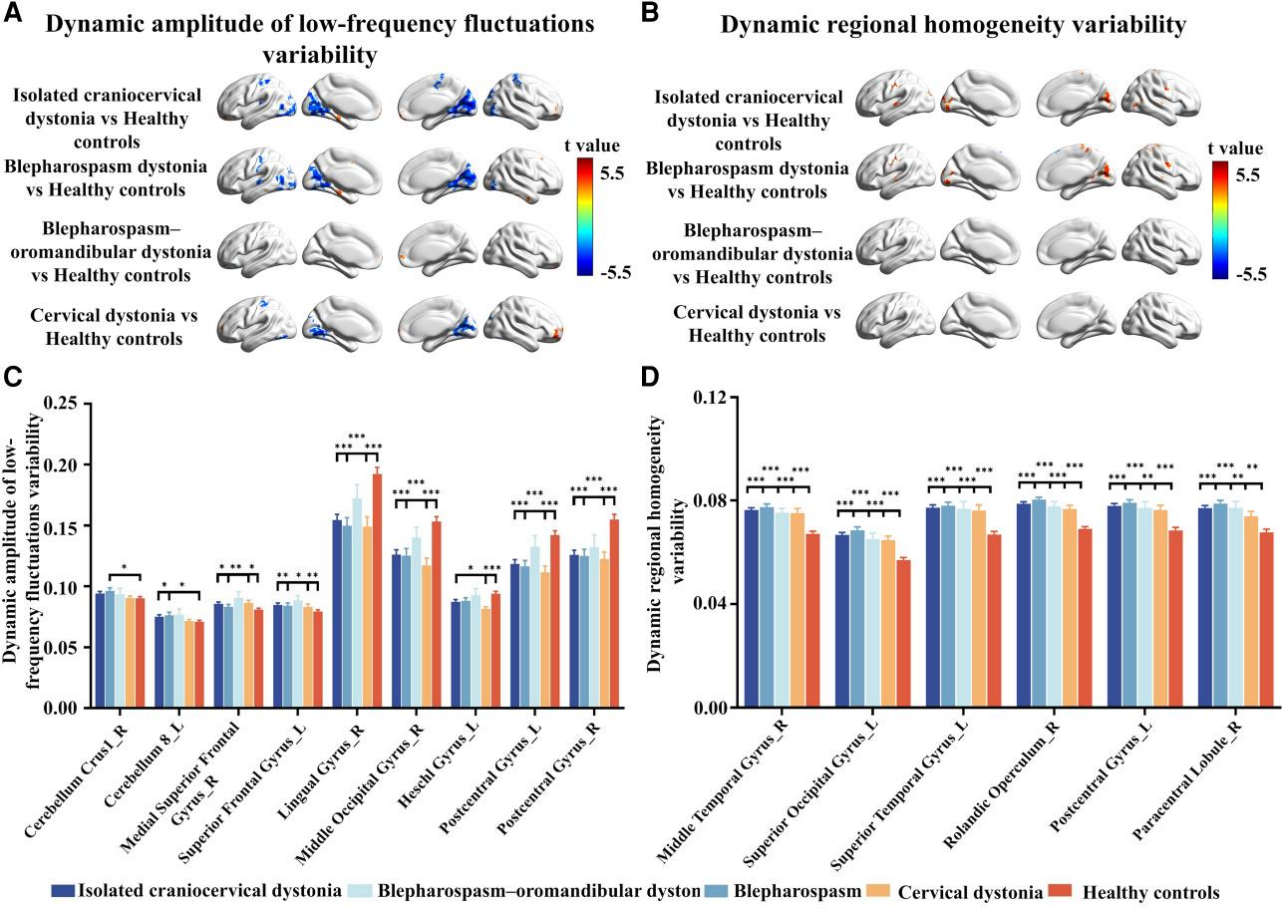
**Brain regions showing significant between-group differences in dALFF variability and dReHo variability, along with the corresponding quantitative analyses.** (**A** and **B**) Brain regions showing significant differences in dALFF and dReHo variability between dystonia subtypes (CCD, *n* = 201; BSP, *n* = 102; BOM, *n* = 43; CD, *n* = 56) and healthy controls (HCs, *n* = 160). Group differences were compared using two-sample *t*-tests. The colour bar represents *t*-values, with warm colours indicating increased variability and cold colours indicating decreased variability. The statistical threshold was set at a voxel-level of *P* < 0.01 and a cluster-level of *P* < 0.05, corrected using GRF theory. (**C** and **D**) Within the significant brain regions identified in the comparison between CCD (*n* = 201) and HCs (*n* = 160), *post hoc* group differences in dALFF and dReHo variability were further assessed using two-sample *t*-tests and corrected using the FDR. Asterisks indicate statistically significant group differences (*P < 0.05, **P < 0.01, ***P < 0.001).

Compared to HCs (Fig. 2A and C and Supplementary Table 1), CCD patients showed significantly increased dALFF variability in the right cerebellum crus I, right medial superior frontal gyrus, left cerebellum 8 and left superior frontal gyrus (GRF corrected, a voxel level *P* < 0.01, a cluster level *P* < 0.05), and significantly decreased dALFF variability in the right lingual gyrus, right middle occipital gyrus, left heschl's gyrus and bilateral postcentral gyrus (GRF corrected, a voxel level *P* < 0.01, a cluster level *P* < 0.05).

Compared to HCs (Fig. 2B and D and Supplementary Table 1), the CCD group exhibited significantly increased dReHo in the right middle temporal gyrus, left superior occipital gyrus, left superior temporal gyrus, right rolandic operculum, left postcentral gyrus and right paracentral lobule (GRF corrected, a voxel level *P* < 0.01, a cluster level *P* < 0.05).

BSP, BOM and CD patients displayed similar variability in cortical regions as CCD patients, with BSP showing higher dALFF variability in the right caudate (Fig. 2A–D and Supplementary Table 1).

### Between-group differences in dFC variability

**Figure 3:**
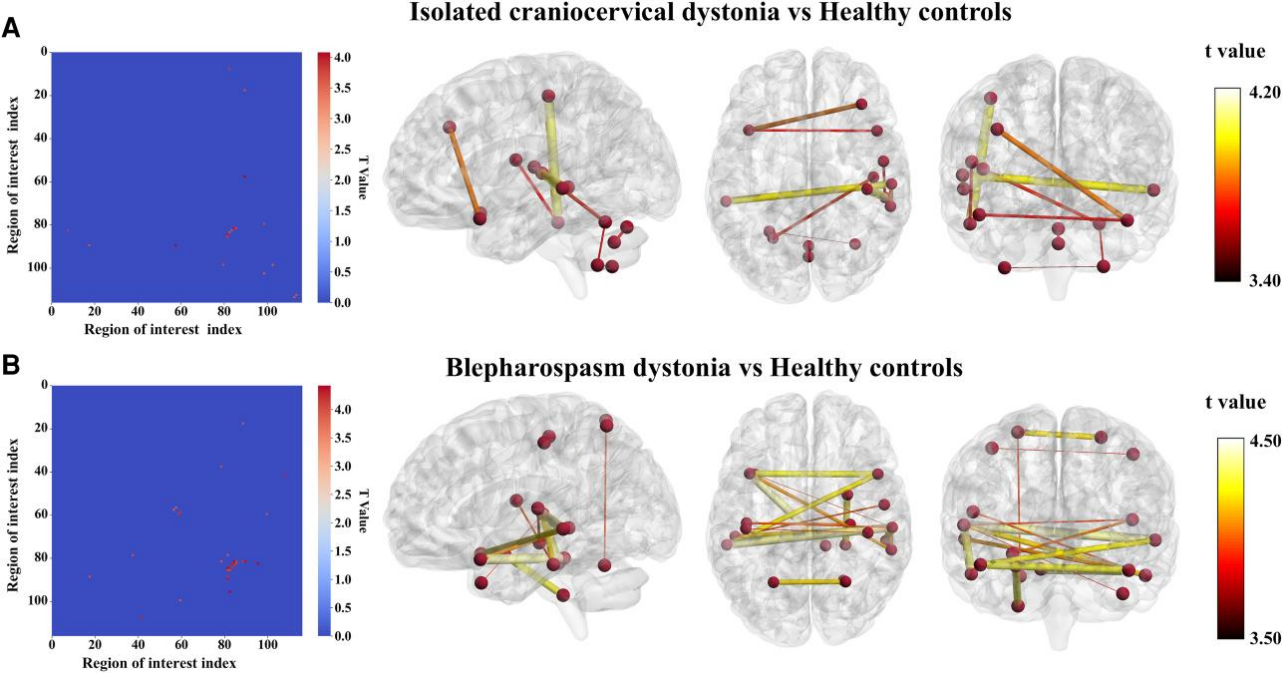
**Differences in functional connectivity variance between (A) isolated craniocervical dystonia (CCD, *n* = 201) and healthy controls (HCs, *n* = 160), and between (B) blepharospasm dystonia (BSP, *n* = 102) and healthy controls (HCs, *n* = 160).** Group differences were compared using two-sample *t*-tests, and the results were corrected using NBS (edges with *P* < 0.005 and components with *P* < 0.05). The left panels display the functional connectivity variance matrices, whereas the top 10% of *t*-values were retained, and all remaining values were set to zero. The horizontal and vertical axes represent brain regions numbered according to the AAL116 template. The right panels show the 3D brain maps, whereas nodes and edges are colour-coded based on the retained *t*-value, with higher *t*-values indicating stronger connectivity differences.

After multiple comparisons correction using NBS (edge *P* < 0.005, component *P* < 0.05, iteration = 1000) and two-sample *t*-test, no significant differences in dFC variability between the BOM\CD groups and HCs were identified. However, significant changes in connectivity sub-networks were observed between the CCD\BSP and HCs. Figure 3A and B and Table 2 display the top 10% of significant brain regions in the FC variability matrix with the highest connection strength in different group comparisons. Compared to HCs, the CCD group showed significantly increased dFC variability between the right middle frontal gyrus and left superior temporal pole, right rolandic operculum and right inferior temporal gyrus, right postcentral gyrus and right inferior temporal gyrus, right heschl’s gyrus and left cerebellum 6, right superior temporal gyrus and left middle temporal gyrus, right superior temporal gyrus and right middle temporal gyrus, left superior temporal pole and right superior temporal pole, left cerebellum 6 and left cerebellum 8, right cerebellum 7b and left cerebellum 8 and vermis 7 and vermis 8. In addition, we found significant enhancement of dFC variability between cortical regions and cerebellar regions in BSP patients.

**Table 2:** Comparison of the top 10% brain regions with the most significant functional connectivity variance differences between HCs, CCD and BSP groups

Pathways	*t*-value	*P*-value
**CCD** **>** **HCs**		
Middle Frontal Gyrus_R & Superior Temporal Pole_L	3.826	<0.001
Rolandic Operculum_R & Inferior Temporal Gyrus_R	3.693	<0.001
Postcentral Gyrus_R & Inferior Temporal Gyrus_R	4.079	<0.001
Heschl’s Gyrus_R & Cerebellum 6_L	3.726	<0.001
Superior Temporal Gyrus_R & Middle Temporal Gyrus_L	3.990	<0.001
Superior Temporal Gyrus_R & Middle Temporal Gyrus_R	3.796	<0.001
Superior Temporal Pole_L & Superior Temporal Pole_R	3.686	<0.001
Cerebellum 6_L & Cerebellum 8_L	3.626	<0.001
Cerebellum 7b_R & Cerebellum 8_L	3.555	<0.001
Vermis 7 & Vermis 8	3.676	<0.001
**BSP** **>** **HCs**		
Rolandic Operculum_R & Inferior Temporal Gyrus_L	3.981	<0.001
Hippocampus_R & Heschl’s Gyrus_L	4.022	<0.001
Amygdala_R & Cerebellum 10_R	4.300	<0.001
Postcentral Gyrus_L & Postcentral Gyrus_R	3.910	<0.001
Superior Parietal Lobule_L & Superior Parietal Lobule_R	4.193	<0.001
Superior Parietal Lobule_R & Cerebellum 6_R	3.961	<0.001
Heschl’s Gyrus_L & Superior Temporal Gyrus_R	3.968	<0.001
Middle Temporal Gyrus_L & Superior Temporal Gyrus_R	4.411	<0.001
Middle Temporal Gyrus_R & Superior Temporal Gyrus_R	4.086	<0.001
Middle Temporal Pole_L & Superior Temporal Gyrus_R	3.908	<0.001
Inferior Temporal Gyrus_L & Superior Temporal Gyrus_R	4.177	<0.001
Inferior Temporal Gyrus_R & Superior Temporal Gyrus_R	4.359	<0.001
Superior Temporal Pole_R & Superior Temporal Pole_L	4.287	<0.001
Middle Temporal Gyrus_R & Superior Temporal Pole_L	4.094	<0.001
Cerebellum 3_R & Superior Temporal Pole_L	4.422	<0.001
Superior Temporal Pole_R & Middle Temporal Gyrus_L	4.211	<0.001

Abbreviations: BSP, blepharospasm; CCD, isolated craniocervical dystonia; HCs, healthy controls; L, left; R, right.

### Between-group differences in the temporal properties of FC states

**Figure 4:**
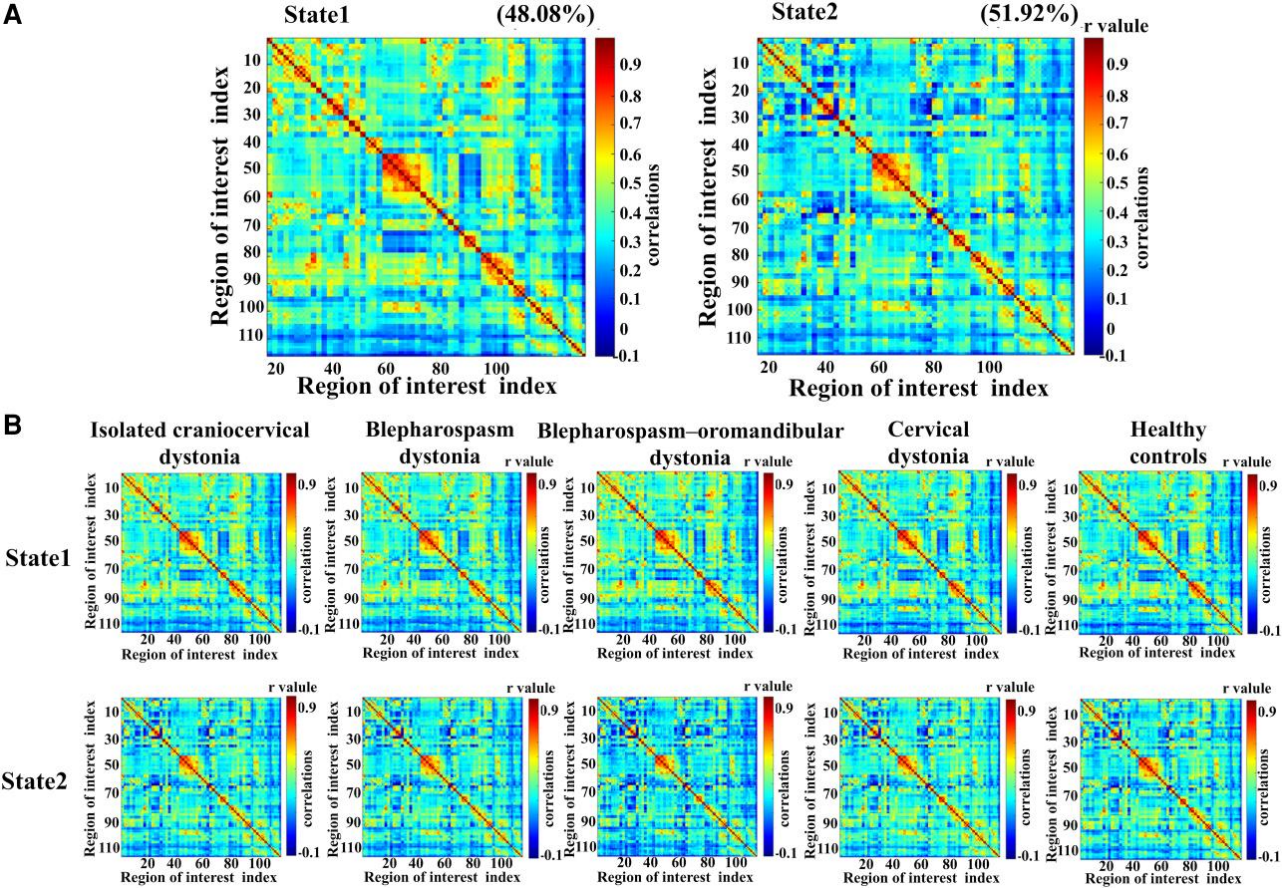
**Two dynamic functional networks were identified through clustering.** (**A**) The two cluster centroids from all sliding time windows of all subjects (*n* = 361) are shown, along with their occurrence percentages. State 1 occurred in 48.05% of the time windows, primarily characterized by stronger interactions, whereas State 2 occurred in 51.92% of the time windows, primarily characterized by weaker interactions. (**B**) Functional connectivity state results. Group centroid matrices for each state are as follows: the total occurrence percentages for State 1 and State 2 were 43.23% and 56.77% for the isolated craniocervical dystonia group (CCD, *n* = 201), 44.44% and 55.56% for the blepharospasm dystonia group (BSP, *n* = 102), 47.62% and 52.38% for the blepharospasm–oromandibular dystonia group (BOM, *n* = 43), 33.26% and 66.84% for the cervical dystonia group (CD, *n* = 56), and 53.73% and 46.27% for the healthy controls group (HCs, *n* = 160). The horizontal and vertical axes represent brain regions numbered according to the AAL116 template.

We applied the K-means clustering method and identified two recurring state patterns (State 1 and State 2), as shown in Fig. 4A. State 1 exhibits a lower occurrence frequency (48.08%), with relatively stronger connections in the brain network. State 2 shows a higher occurrence frequency (51.92%), with relatively weaker connections in the brain network. Figure 4B presents the state- and group-specific cluster centres derived from K-means clustering analysis. In State 1, compared to the HCs, CCD patients displayed enhanced dFC strength between the left olfactory region and left cerebellum 10 (*P* < 0.01, top 0.4%, Supplementary Table 2). Additionally, CCD patients exhibited reduced dFC strength between the right rolandic operculum and right postcentral gyrus, right hippocampus and left parahippocampal gyrus and right middle temporal gyrus and right temporal pole (*P* < 0.01, top 0.4%). In State 2, compared to the HCs, CCD patients showed increased dFC strength between the right thalamus and right inferior temporal gyrus (*P* < 0.01, top 0.4%). Furthermore, CCD patients demonstrated weaker dFC strength between the right rolandic operculum and left Heschl gyrus, right Heschl gyrus and left superior temporal gyrus and Vermis 7 and Vermis 9 (*P* < 0.01, top 0.4%). BSP, BOM and CD patients exhibited characteristics similar to CCD patients in both State 1 and State 2. Moreover, compared to the HCs, BSP patients showed decreased dFC variability between the left putamen and right pallidum, while BOM patients displayed increased variability in the left middle cingulate gyrus and vermis 6.

**Figure 5:**
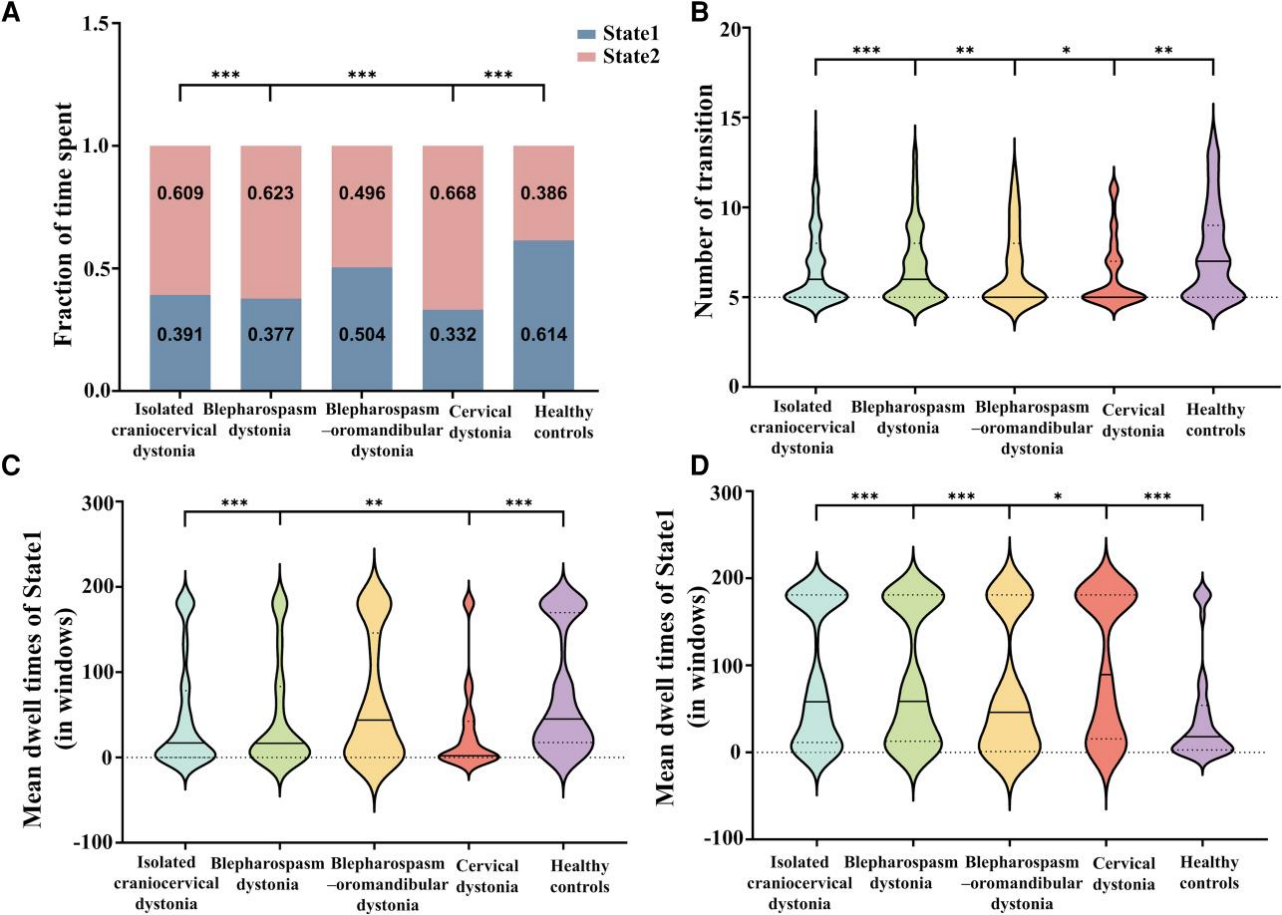
**Comparisons of the temporal properties of dFC states among the isolated craniocervical dystonia group (CCD, *n* = 201), blepharospasm dystonia group (BSP, *n* = 102), blepharospasm–oromandibular dystonia group (BOM, *n* = 43), cervical dystonia group (CD, *n* = 56) and healthy controls group (HCs, *n* = 160) were performed using two-sample *t*-tests, with results corrected using FDR.** (**A**) Percentage of total time spent in each state. (**B**) Number of transitions and (**C** and **D**) MDT were plotted using violin plots. Horizontal lines indicate group medians and interquartile range (solid and dashed lines, respectively). *P < 0.05, **P < 0.01, ***P < 0.001.

Despite the similarity in dFC patterns between these two states, abnormalities in the temporal properties of different patient subtypes were observed. As shown in Fig. 5A, the HCs group had a total occurrence rate of 60.9 ± 37.6% in State 1 and 39.1 ± 37.6% in State 2. All four patient groups exhibited a higher occurrence frequency in State 2 and a lower frequency in State 1 compared to the HCs (*P* < 0.05, FDR corrected). Regarding state transition frequency (Fig. 5B), the HCs group showed the highest frequency of transitions between the two states, whereas all four patient groups displayed significantly lower transition frequencies (*P* < 0.05, FDR corrected). Furthermore, all four patient groups spent more time in State 2, with a relatively shorter average dwell time in State 1 compared to the HCs (*P* < 0.05, FDR corrected, Fig. 5C and D).

### Correlations between dALFF, dReHo, dFC temporal properties and clinical characteristics

No significant correlations were found between the altered dALFF variance, dReHo variance, dFC temporal properties and the JRS severity subscore, JRS frequency subscore and JRS total score in the BSP, or disease duration in the CCD, BSP, BOM and CD groups.

## Discussion

This study utilized dALFF and dReHo to elucidate differences in resting-state brain activity between patients with CCD\BSP\BOM\CD and HCs. The findings revealed widespread abnormalities in dynamic brain activity within cortical and cerebellar regions in these patients. Further analysis using dFC demonstrated variability in dFC strength, with similar patterns of dynamic functional network connectivity across different subtypes of dystonia. Clustering analysis showed two structured states: State 1 characterized by stronger dFC, and State 2 characterized by weaker dFC. Compared to HCs, the CCD\BSP\BOM\CD group exhibited longer dwell time in State 2 and reduced state transition frequency. In State 2, CCD patients displayed dynamic dysfunctional connectivity in the cortico-thalamo-cerebellar circuit. In contrast, in State 1, enhanced dFC was observed between the left olfactory cortex and left Cerebellum 10, whereas reduced dFC was noted between the right Rolandic operculum and right postcentral gyrus. These findings provide novel insights into the neurophysiological regulation of CCD and offer potential targets for therapeutic strategies in CCD.

The current study indicates that BSP/BOM/CD and CCD subjects exhibit both shared and specific dynamic features in brain activity and connectivity. Specifically, BSP and CCD subjects show significantly increased dALFF in certain cerebellar regions and decreased dALFF in the right lingual gyrus and left postcentral gyrus. Additionally, these subjects display significantly increased dReHo in the left superior occipital gyrus, left superior temporal gyrus and left postcentral gyrus. BOM and CCD subjects exhibit increased dALFF variability in frontal regions, whereas CD and CCD subjects show significantly increased dALFF in the left superior frontal gyrus, as well as significantly decreased dALFF in the right lingual gyrus and left postcentral gyrus. Notably, dALFF and dReHo reflect the temporal variability of brain activity intensity and local synchronization, respectively.^40,41^ A decrease in both (excessive stability) indicates functional impairment, while an increase in both (excessive variability) suggests instability in neural activity or coordination.^42,43^ These findings reveal that BSP/BOM/CD patients may exhibit asymmetric brain function impairment and disrupted neural activity. By comparing the dALFF and dReHo variability values of CCD, BSP, BOM and CD with those of HC, it is evident that different dystonia subtypes share similarities in dynamic functional characteristics, suggesting they may share common neurophysiological mechanisms.

According to traditional views, the basal ganglia are considered as the primary brain structure involved in the pathophysiology of dystonia.^44^ However, recent animal studies have shown that experimental manipulation of the cerebellum leads to dystonia-like motor manifestations.^45^ Another animal study observed that pharmacological excitation of the cerebellum can lead to dystonia.^46^ Furthermore, several clinical observations, including secondary dystonia cases and neurophysiological and neuroimaging studies of human patients, provide further evidence of a potential link between cerebellar abnormalities and dystonia.^46-48^ Based on these views, our findings of significantly increased dALFF in the right cerebellum Crus I, left cerebellum 8, and right cerebellum 7b in CCD and BSP groups are consistent with the idea that dysfunction in these regions may be key factors in the onset of motor symptoms in dystonia patients, highlighting the importance of the cerebellum in the motor regulation pathology of dystonia.^49^ Although no significant changes in cerebellar dALFF or dReHo were observed in BOM and CD patients in the present study, previous studies have indicated that the cerebellum may still play an important role in these subtypes. For example, in CD, multiple neuroimaging and neurophysiological studies have demonstrated abnormalities in the cerebello-thalamo-cortical pathway, grey matter structural changes and reduced cerebellar inhibition, with these alterations correlated with symptom severity^50,51^; in BOM, functional neuroimaging studies have similarly revealed cerebellar metabolic abnormalities and altered connectivity patterns.^45^ These findings suggest that network-level dysregulation involving the cerebellum may still constitute an important pathophysiological mechanism in subtypes such as BOM and CD.^52^

The visual pathway includes the ventral and dorsal streams. The ventral stream originates in the primary visual cortex (V1) and projects to the inferior temporal cortex. The lingual gyrus is a key node in the ventral stream, often referred to as the ventral occipito-temporal area (including the lingual gyrus, fusiform gyrus and parahippocampal gyrus), which is involved in object recognition.^53^ In this study, we found that dALFF in the right lingual gyrus in BSP and CD groups and in the left superior occipital gyrus in BSP patients was significantly reduced, which may be associated with the ventral visual stream's function in processing facial and other complex visual information. This, in turn, may reduce the reliance on visual feedback during action execution or posture adjustment, exacerbating abnormal postures or involuntary movements.

Our analysis showed no significant difference in dFC variability between the BOM/CD and HCs groups, but we observed similar patterns of dFC variability in CCD and BSP patients. Compared to HCs, we found increased dFC variability in sensory-motor regions, the temporal lobe and cerebellar networks in dystonia patients, with widespread connectivity disruptions. This is consistent with previous neuroimaging studies emphasizing the role of cortico-cortical and cortico-cerebellar circuits in dystonia.^54,55^ These results suggested that dFC can provide more subtle dynamic brain function characteristics on a temporal scale. Notably, the increased dFC between the right postcentral gyrus and right inferior temporal gyrus, as well as between the right superior temporal gyrus and bilateral middle temporal gyrus, may reflect compensatory mechanisms for dystonic symptoms. They attempted to alleviate motor abnormalities by enhancing the connectivity between the somatosensory cortex and the temporal lobe network, leading to maladaptive network remodelling and exacerbating symptom heterogeneity.^4^

The whole-brain network dFC can be clustered into two recurring states: one state is more frequent with weaker connections (State 1), and the other is less frequent with stronger connections (State 2), indicating that FC is not fixed, but changes over time. In our study, CCD patients showed increased dFC between the right thalamus and right inferior temporal gyrus, and a greater proportion of time spent in State 2 with decreased dFC variability in the Vermis 7 and Vermis 9, right Heschl gyrus and left superior temporal gyrus, as well as right rolandic operculum and left Heschl gyrus. These results were consistent with the key role of the cortico-thalamo-cerebellar circuit in dystonia pathology.^56^ The dynamic dysregulation of the cerebellum and thalamus suggests overall dysfunction of the cortico-thalamo-cerebellar loop. Developmental abnormalities in cerebellar output pathways may be the cause of the local metabolic abnormalities observed at rest, potentially leading to altered cortical activation responses during motor tasks and learning, affecting cortical plasticity and causing defects in sensory-motor integration, which aligns with previously reported microstructural defects in cerebellar white matter.^57^

In State 1, we observed increased dFC variability between the left olfactory and left cerebellum 10. The dysfunction of olfactory has been identified as a common non-motor symptom in neurodegenerative and movement disorders.^58^ Recent genetic studies have found missense and nonsense mutations in the GNAL gene in dystonia families through whole-exome sequencing, which mainly cause adult-onset cervical and segmental dystonia.^59,60^ GNAL encodes the Gαolf protein, which is highly expressed in the striatum and cerebellar Purkinje cells, and is also identified as the G protein that mediates odour signals in olfactory neuroepithelium.^61^ Additionally, several fMRI and PET-based human functional imaging studies have shown specific cerebellar activation during olfactory recognition and discrimination tasks.^62^ Therefore, the increased dFC variability between the left olfactory and left cerebellum 10 may be related to molecular mutations associated with Gαolf, and the dysregulation of the olfactory-cerebellar loop may lead to ineffective compensation, affecting the efficiency of odour signal integration, which aligns with the clinical presentation of olfactory dysfunction.^63^ We also found that the decreased dFC in State 2 between the right rolandic operculum and right postcentral gyrus reflects neural changes leading to abnormal sensory-motor processing, potentially related to the mechanism by which sensory skills attempt to alleviate symptoms through modulation of cortical-cortical connectivity.^64^

Furthermore, our study found that CCD exhibited a reduced number of state transitions during the resting state. This phenomenon is consistent with findings reported in other neurological disorders (e.g. Alzheimer’s disease and epilepsy) and psychiatric disorders (e.g. schizophrenia), suggesting that decreased dynamic flexibility of brain networks may represent a transdiagnostic feature.^65-67^ Previous studies have further shown that prolonged dwelling time in weakly connected states and fewer state transitions are associated with impaired striatal dopaminergic function (e.g. as indicated by dopamine transporter imaging), which aligns with our results and implies that alterations in large-scale functional dynamics may be related to dopaminergic loss.^24^ Meanwhile, studies on medication effects have also shown that dopamine replacement therapy can enhance neural network flexibility, as reflected by more frequent state transitions.^68^ From a functional perspective, dynamic switching among different connectivity states may enable the brain to mobilize the necessary resources more rapidly in response to current demands, whereas lower network variability may reflect reduced system complexity, thereby diminishing the ability to effectively adapt to environmental changes or unexpected stimuli.^69^ Taken together, the reduced transition counts and the increased time spent in the weakly connected State 2 in the CCD group indicate a relatively ‘rigid’ brain network dynamic; such weak connectivity states typically correspond to reduced network activity or increased segregation between modules, which may limit information transmission and integration and, in turn, be associated with impaired cognitive flexibility.^16^

This study has several limitations. First, given the frequent comorbidity of anxiety and depression in dystonia, incomplete neuropsychological data in our cohort limited further analyses of associations between non-motor symptoms and imaging metrics. Therefore, future studies should incorporate more comprehensive non-motor assessments to better delineate their links with dynamic brain functional alterations; thus, the underlying neural mechanisms of these symptoms require further investigation. Second, the selection of window size in sliding-window analysis remains controversial, and the optimal size for capturing dynamic brain activity is unknown; future studies should validate the present findings using different window lengths. Lastly, although significant changes in dFC variability were observed in the CCD and BSP groups, no significant differences emerged between the BOM/CD group and HCs. This subtype-specific discrepancy may stem from greater internal heterogeneity, smaller effect sizes and limited sample sizes in the BOM/CD subtypes.^52,70^ Moreover, abnormalities in BOM/CD may primarily associate with specific pathways or local nodes rather than whole-brain dFC variability.^71^ Future studies with larger subtype-specific samples and targeted pathway analyses will help clarify the neural mechanisms of these inter-subtype differences.

## Conclusion

Our findings reveal that CCD patients exhibit impaired functional activity in cerebellar-cortical and cortical-cortical circuits, and that different dystonia subtypes display both shared and subtype-specific abnormalities in dynamic brain activity and FC patterns, as assessed by dynamic functional metrics and dFC. These results suggest that the pathophysiological mechanisms of dystonia are associated with dynamic instability in neural networks, functional abnormalities, and network reorganization in specific brain regions. These findings may provide more comprehensive insights into the pathophysiological mechanisms of CCD and facilitate the development of therapeutic strategies.

## Supplementary Material

fcag122_Supplementary_Data

## Data Availability

All data supporting the findings of this study are available within the article and its Supplementary Information files. The data that support the findings are available from the corresponding author upon reasonable request. The dALFF and dReHo variability analyses were performed using DPABI software (https://rfmri.org/DPABI), while dynamic functional connectivity analysis was conducted using DynamicBC software (https://github.com/guorongwu/DynamicBC). Brain network connectivity was visualized using BrainNet Viewer (https://www.nitrc.org/projects/bnv/). Network-Based Statistic correction and two-sample *t*-tests were performed using the GRETNA toolbox (www.nitrc.org/projects/gretna).
