# Supplementary material for: Shared and distinct dynamic brain activity and functional connectivity variability in patients with isolated craniocervical dystonia
Source: Brain Commun. 2026 Apr 2;8(3):fcag122. doi: 10.1093/braincomms/fcag122 (PMC13184622; doi:10.1093/braincomms/fcag122)
Supplement: fcag122_Supplementary_Data [file fcag122_supplementary_data.pdf]

Supplementary Table 1 Brain regions showing significant differences in dALFF and dReHo variance between groups

Groups	Region	Side	Voxel size,mm3	Peak MNI coordinate			Peak intensity
				X	Y	Z	
**Differences in dALFF variability**							
CCD>HCs	Cerebellum Crus I	R	31	27	-75	-27	4.134
	Cerebellum 8	L	35	-30	-60	-48	3.967
	Medial Superior Frontal Gyrus	R	58	18	45	6	4.972
	Superior Frontal Gyrus	L	81	-18	36	-12	4.289
CCD<HCs	Lingual Gyrus	R	282	12	-78	-6	-5.436
	Middle Occipital Gyrus	R	47	42	-78	3	-4.291
	Heschl's Gyrus	L	21	-36	-30	12	-4.899
	Postcentral Gyrus	L	85	-51	-30	57	-4.562
	Postcentral Gyrus	R	130	36	-33	69	-4.311
BSP>HCs	Cerebellum 7b	R	32	24	-78	-51	4.283
	Inferior Temporal Gyrus	R	65	48	-9	-33	4.995
	Parahippocampal Gyrus	L	24	-30	-24	-18	4.417
	Caudate	R	68	21	21	12	4.937
BSP<HCs	Middle Temporal Gyrus	R	38	-60	-21	0	-3.930
	Lingual Gyrus	R	216	12	-81	-6	-4.921
	Inferior Occipital Gyrus	R	46	42	-81	-9	-4.226
	Postcentral Gyrus	L	35	-51	-30	57	-3.850
BOM>HCs	Middle Frontal Gyrus, Orbital Part	L	32	-27	66	-9	4.651
	Middle Frontal Gyrus, Orbital Part	R	28	6	51	-30	3.763
CD>HCs	Rectus Gyrus	R	19	3	48	-24	4.553
	Middle Frontal Gyrus	R	37	24	48	6	5.407
	Superior Frontal Gyrus	L	14	-24	54	6	4.327
CD<HCs	Inferior Occipital Gyrus	L	36	-42	-78	-9	-4.009
	Inferior Occipital Gyrus	R	23	39	-72	-12	-3.757
	Lingual Gyrus	R	160	18	-57	-6	-4.877
	Postcentral Gyrus	L	72	-36	-33	54	-4.215
**Differences in dReHo variability**							
CCD>HCs	Middle Temporal Gyrus	R	27	45	-72	0	3.635
	Superior Occipital Gyrus	L	29	-9	-99	18	4.859
	Superior Temporal Gyrus	L	32	-66	-21	6	4.370
	Rolandic Operculum	R	34	54	-9	21	4.746
	Postcentral Gyrus	L	46	-42	-15	36	3.968
	Paracentral Lobule	R	7	0	-33	63	3.575
BSP>HCs	Calcarine Cortex	R	81	9	-81	15	4.774
	Superior Temporal Gyrus	L	23	-66	-27	12	3.745
	Postcentral Gyrus	R	64	54	-9	33	4.983
	Superior Occipital Gyrus	L	19	-9	-99	18	4.628
	Postcentral Gyrus	L	54	-45	-15	36	4.604
	Superior Parietal Lobule	R	15	24	-51	60	4.315

Abbreviations: BSP, blepharospasm; CCD, Isolated craniocervical dystonia; HCs, healthy controls; CD, craniocervical dystonia; MNI, Montreal Neurological Institute standard space; BOM, blepharospasm-oromandibular dystonia; dALFF, dynamic amplitude of low-frequency fluctuations; dReHo, dynamic regional homogeneity; L, left; R, right.

Supplementary Table 2 In each state, the top 0.4% of the most significant strong connections were preserved

Pathways	t value	p value	Pathways	t value	p value
**Differences in dFC variability of state 1**			**Differences in dFC variability of state 2**		
**CCD>HCs**			**CCD>HCs**		
Olfactory_L & Cerebellum 10_L	2.379	0.018	Rolandic Operculum_R & Heschl Gyrus_L	-4.530	>0.001
**CCD<HCs**			Heschl Gyrus_R & Superior Temporal Gyrus_L	-4.614	>0.001
Rolandic Operculum_R & Postcentral Gyru_R	-3.888	>0.001	Vermis7 & Vermis9	-4.387	>0.001
Hippocampus_R & Parahippocampal Gyrus_L	-3.868	>0.001	**CCD<HCs**		
Middle Temporal Gyrus_R & Temporal Pole Middle Temporal Gyrus_R	-4.052	>0.001	Thalamus_R & Inferior Temporal	3.427	>0.001
**BSP>HCs**			**BSP>HCs**		
Precentral Gyrus_L & Cerebellum Crus 1_R	2.437	>0.001	Angular Gyrus_R & Cerebellum 4_5_R	2.607	0.010
**BSP<HCs**			**BSP<HCs**		
Rolandic Operculum_R & Postcentral Gyrus_R	-4.588	>0.001	Putamen_L & Putamen_R	-4.758	>0.001
Hippocampus_R & Parahippocampal Gyrus_L	-4.308	>0.001	Putamen_L & Pallidum_R	-5.012	>0.001
Middle Temporal Gyrus_R & Temporal Pole Middle Temporal Gyrus_R	-4.415	>0.001	Pallidum_L & Pallidum_R	-4.907	>0.001
**BOM>HCs**			**BOM>HCs**		
Precuneus_L & Vermis12	2.886	0.004	Paracentral Lobule_L & Caudate Nucleus_R	2.927	0.004
**BOM<HCs**			**BOM<HCs**		
Superior Frontal Gyrus Orbital Part Medial _L & Anterior Cingulate Gyrus_L	-4.429	>0.001	Middle Cingulate Gyrus_L & Vermis6	-4.545	>0.001
Cerebellum 6 _R & Cerebellum 8_L	-4.548	>0.001	Middle Cingulate Gyrus_R & Vermis6	-4.886	>0.001
**CD>HCs**			Vermis7 & Vermis8	-5.163	>0.001
Olfactory_L & Cerebellum 10 L	4.177	>0.001	Vermis7 & Vermis9	-4.890	>0.001
**CD<HCs**			Middle Occipital Gyrus_L & Postcentral Gyrus_R	-3.573	>0.001
Supramarginal Gyrus _L & Supramarginal Gyrus_R	-4.071	>0.001	**CD>HCs**		
Cerebellum 6 _R & Cerebellum 8_L	-4.548	>0.001	Olfactory_R & Temporal Pole Middle Temporal Gyrus_L	3.394	>0.001
			Superior Occipital Gyrus_R & Cerebellum 10_L	3.689	>0.001
			**CD<HCs**		
			Middle Occipital Gyrus_L & Postcentral Gyrus_R	-3.573	>0.001

Abbreviations: BSP, blepharospasm; CCD, Isolated craniocervical dystonia; HCs, healthy controls; CD, craniocervical dystonia; BOM, blepharospasm-oromandibular dystonia; dFC, dynamic functional connectivity; L, left; R, right.
